# Intraoperative Fluorescence Imaging of Peripheral and Central Nerves Through a Myelin-Selective Contrast Agent

**DOI:** 10.1007/s11307-012-0555-1

**Published:** 2012-04-10

**Authors:** Victoria E. Cotero, Tiberiu Siclovan, Rong Zhang, Randall L. Carter, Anshika Bajaj, Nicole E. LaPlante, Evgenia Kim, Daniel Gray, V. Paul Staudinger, Siavash Yazdanfar, Cristina A. Tan Hehir

**Affiliations:** GE Global Research, One Research Circle, Niskayuna, NY 12309 USA

**Keywords:** Image-guided surgery, Nerves, Myelin, Fluorescence, Compact instrumentation

## Abstract

**Purpose:**

Patients suffer from complications as a result of unintentional nerve damage during surgery. We focus on improving intraoperative visualization of nerves through the use of a targeted fluorophore and optical imaging instrumentation.

**Procedure:**

A myelin-targeting fluorophore, GE3111, was synthesized, characterized for its optical and myelin-binding properties using purified myelin basic protein, and evaluated in mice. Additionally, a compact instrument was adapted to visualize nerves.

**Results:**

GE3111 was synthesized using a versatile methodology. Its optical properties were sensitive to the local environment both *in vitro* and *in vivo*. Following intravenous injection, central and peripheral nerves were visualized, with the kinetics of nerve uptake modifiable depending on the formulation. Fluorescence polarization showed specific and strong binding to purified myelin basic protein. Nerves were visualized *in vivo* using a dedicated compact imaging device requiring less than 2.5 mW/cm^2^ of illumination at 405 nm.

**Conclusions:**

Fluorescence imaging of nerves through myelin showed a potential for use in image-guided surgery. Intraoperative nerve imaging is an example where contrast agent and instrument development come together as a result of clinical need.

**Electronic supplementary material:**

The online version of this article (doi:10.1007/s11307-012-0555-1) contains supplementary material, which is available to authorized users.

## Introduction

Iatrogenic damage to peripheral nerves is a major cause of morbidity associated with many surgical procedures, including prostatectomy [1–4], coronary artery bypass graft [5–7], thyroidectomy [8, 9], rhytidectomy [10], and breast cancer surgery [11–13]. Symptoms associated with nerve damage are dependent upon the location, type of nerve, and the severity of the damage, and may result in loss of function, weakness, muscle atrophy, fasciculation, paralysis, cardiac irregularities, allodynia, and chronic neuropathy [14]. The cause of nerve damage during open and laparoscopic surgical procedures is variable but is often the result of inadvertent surgical damage due to poor visibility of the nerve as compared to surrounding tissues or an unfortunate necessity due to close proximity of the nerve to target structures [15]. Currently, most surgical procedures are performed without image guidance, as available technologies lack the specificity needed to provide nerve-selective imaging [16]. Applied nerve-sparing procedures generally rely on anatomical landmark identification and are highly dependent on the surgeon's skill and experience. In addition to visual identification, intraoperative electrical stimulation devices are often employed to verify continued stimulation, via nerve, of the muscle or organ in question [17, 18]. However, there are inherent limitations to relying on these methods alone. Visual identification of nerves can be inconsistent due to the intricacy and size of the individual nerves, and overall variation in the anatomic location across patient populations [19]. Furthermore, intraoperative electrical stimulation fails to prevent nerve damage; rather, it identifies damage that has already occurred. Thus, optical imaging could provide a valuable clinical tool for image-guided surgery by allowing direct and real-time visualization of nerves.

We have previously reported the generation of a nerve-specific fluorophore, 4-[(1E)-2-[4-[(1E)-2-[4-aminophenyl] ethenyl]-3-methoxyphenyl] ethenyl]-benzonitrile (GE3082), that crosses the blood–nerve and blood–brain barriers, producing significant fluorescence in myelinated nerves after a single systemic injection [20]. Because of its lipophilic nature, GE3082 requires a specialized intravenous formulation consisting of 65 % serum, 20 % HEPES, 10 % dimethyl sulfoxide (DMSO), and 5 % Cremophor EL to maintain aqueous solubility, and thus, it is non-ideal for clinical intravenous use due to the potential negative physiologic and pharmacologic effects arising from this formulation [21, 22].

The goal of our study is to advance the current understanding of myelin-targeting fluorophores and to demonstrate *in vivo* imaging of nerves during surgery. We describe here the *in vitro* and *in vivo* characterization of a newly synthesized fluorophore, 1-methylsulfonyl-4-[(1E)-2-[4-[(1E)-2-[4-aminophenyl] ethenyl]-3-methoxyphenyl] ethenyl]-benzene (GE3111). GE3111 was made using a more versatile synthetic methodology with reduced number of steps and more amenable to creating chemical libraries by parallel synthesis. GE3111 had improved aqueous solubility as well as reduced lipophilicity compared with GE3082, allowing for the development of more clinically relevant formulations for intravenous injection. We also describe advancements in the understanding of the myelin-targeting binding interaction, pharmacodynamics, pharmacokinetics, and environmental influences on the optical properties of this fluorophore.

## Materials and Methods

### Synthesis of GE3111

**Fig. 1:**
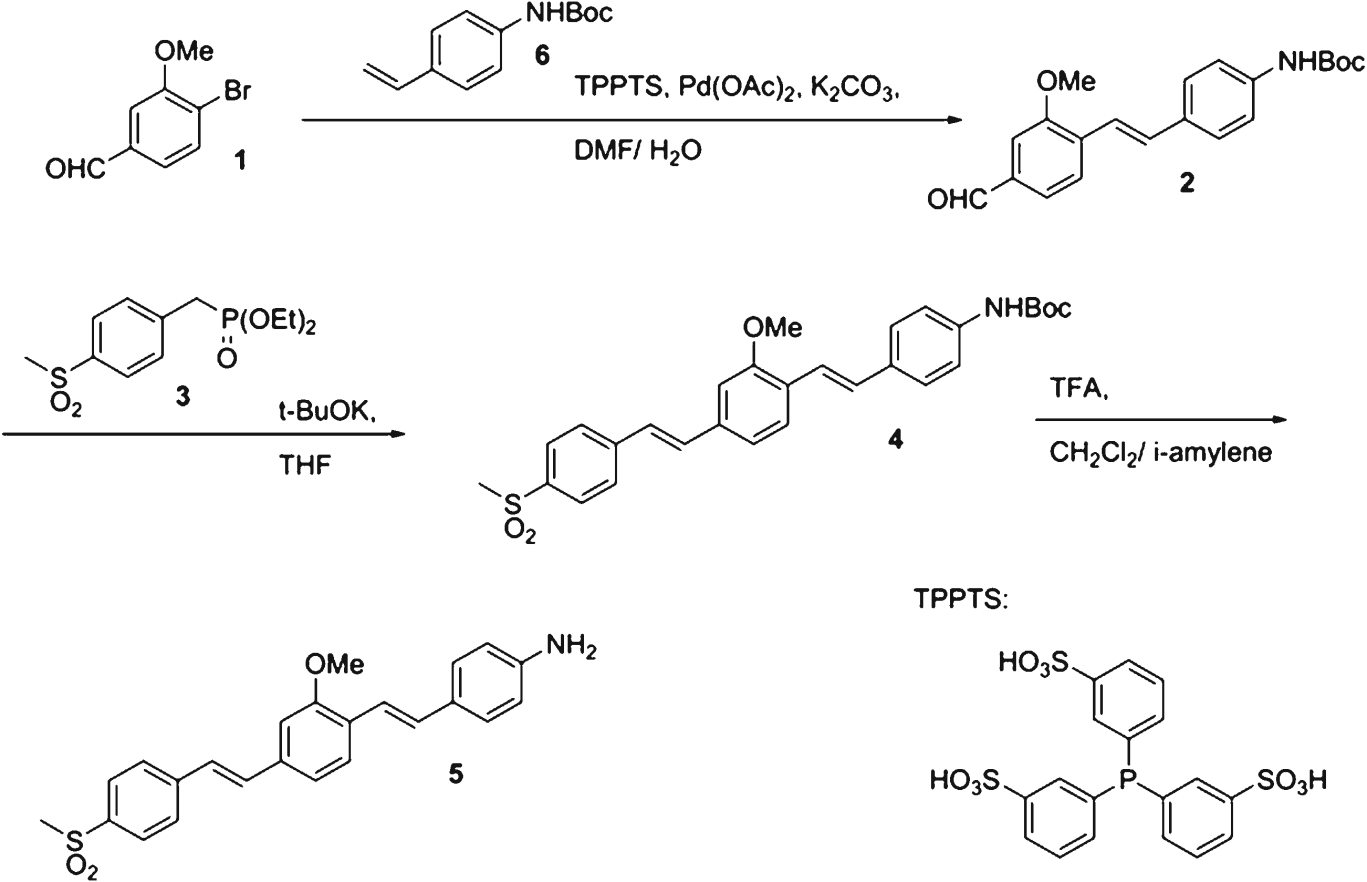
Schematic chemical synthesis of GE3111, a myelin-targeting fluorophore.

GE3111 was synthesized in a stepwise procedure as shown in Fig. 1. Heck coupling [23] of 4-bromo-3-methoxybenzaldehyde [24] with Boc-protected 4-amino styrene in the presence of the water-soluble TPPTS catalyst proceeded in 70 % yield after purification, to give stylbene aldehyde **2**. Subsequent olefination [25] with the phosphonate **3** proceeded in 65 % yield after purification to give the bis-stylbene **4**, exclusively in the trans–trans configuration [26]. Deprotection with trifluoroacetic acid (TFA) in amylene-containing dichloromethane, gave the desired dye **5** in essentially quantitative yield and better than 95 % purity by nuclear magnetic resonance (NMR) spectroscopy. Removal of traces of fluorescent impurities was achieved through a final purification by reverse phase chromatography, eluting with water–acetonitrile gradient containing 0.1 % v/v TFA. The dye was found to be more stable upon storage as its TFA salt; whenever free base dye was needed, a simple aqueous workup (NaHCO_3_/dichloromethane) supplied the required dye as >99.9 % purity. Details of the synthetic methodology can be found in the Supplementary Material.

### Physical and Optical Properties of GE3111

A 10 mM stock solution of GE3111 was prepared in anhydrous dimethylsulfoxide (DMSO) to ensure complete dissolution of the fluorophore. Subsequent aliquots of the stock solution were taken to prepare 10 μM solutions of GE3111 in the following solvents: toluene, olive oil, DMSO, water, and a selected intravenous (IV) formulation (58.5 % distilled water, 30 % 2-hydroxypropyl-β-cyclodextrin, 10 % propylene glycol, 1 % PEG-300, and 0.5 % DMSO). Absorbance spectra were taken using a Lambda 20 UV/Vis spectrometer (Perkin Elmer, Waltham, MA). The wavelength of maximum absorbance was then used as the excitation wavelength for the collection of the fluorescence emission spectra on a steady-state fluorimeter (Photon Technology International, Birmingham, NJ). The molar extinction coefficient (ε) for GE3111 was calculated at the maximum excitation wavelength respective to each solvent, using Beer–Lambert's law. The quantum yield (QY) values of GE3111 in each solvent were measured in comparison to the fluorescence emission of a known standard, coumarin-6 (QY = 78 % [27]), using the single-point method [28].

The logD values of GE3082 and GE3111 at pH 7.4 were calculated using Accelrys Discovery Studio software (San Diego, CA). Maximum solubility was estimated by dissolution of an excess of either GE3111 or GE3082 in formulation (58.5 % distilled water, 30 % 2-hydroxypropyl-β-cyclodextrin, 10 % propylene glycol, 1 % PEG-300, and 0.5 % DMSO), followed by centrifugation (10 min, 10,000 × g). The supernatant was diluted at least 1,000-fold in DMSO, and its absorption spectrum was taken. Concentration was calculated using Beer–Lambert's equation, with *ε* equal to 72,820 and 41,800 M^-1^ cm^-1^ for GE3111 and GE3082, respectively. Dynamic light scattering (DLS) spectroscopy was used to estimate the dispersity of the formulated fluorophores, detailed of which are in the Supplementary Material.

### Myelin Basic Protein Binding Assay

Purified native myelin basic protein (MBP) was obtained from Prof. Paolo Riccio, University of Bari, and was isolated from bovine brain using a published protocol [29]. Details of the fluorescence polarization binding assay will be published elsewhere (Bajaj et al., manuscript in preparation). Briefly, the binding assay reaction was prepared by incubating increasing amounts of native MBP with 100 nM of GE3111 in a 96-well plate. Protein and fluorophore dilutions were made with 0.25 % CHAPS in 20 mM Tris, pH 7.2, which also served as the binding buffer. The reagents were allowed to incubate at room temperature for 10 min; after which, the raw fluorescence intensities parallel (P) and perpendicular (S) to the excitation plane were measured using the fluorescence polarization mode of a Spectra Max M5 plate reader (Molecular Devices, Sunnyvale, CA) at 400 nm excitation and 540 nm emission.

Anisotropy was calculated using the equation = [(*P*−*S*)/(*P*+2*S*)]. Data fitting was performed via non-linear regression using SigmaPlot software (Version 11.2) to obtain the *K*_d_ value.

### *In Vivo* Fluorescence Imaging

#### Instrumentation

*In vivo* imaging consisted of detailed fluorescence emission characterization and surgical imaging using either a Zeiss Lumar imaging system (Carl Zeiss Inc. Thornwood, NY) with coupled multispectral imaging camera (Nuance camera; CRI, Woburn, MA) or a custom compact surgical imaging instrument.

The Zeiss Lumar imaging instrument was used in both the dosing and kinetics studies. A filter centered at 406 nm with a 15-nm bandwidth was used for excitation of the fluorophore. Fluorescence emission data were then recorded at wavelengths ranging from 420 to 720 nm at 10 nm steps using the attached multispectral camera. Fluorescence images were collected using exposure times of 5 s in both control and fluorophore injected animals for normalization. Numerical data presented herein represent the area under the curve for wavelengths ranging from 550 to 720 nm, which mimics our previous study using a 550 longpass filter [20]. This range also included the fluorescence emission maxima for nerve, muscle, and adipose tissue.

Intraoperative imaging was achieved with a custom compact fluorescence imaging instrument modified from the previously developed imaging-guided surgical system [30]. The instrument uses consumer grade cameras and fiber delivery of light to reduce cost and size relative to previous fluorescence instrumentation for open surgical guidance. Various hardware modifications were implemented to accommodate the spectroscopic properties of the fluorophore. The excitation light source was a 500 mW 405 nm laser (Shanghai Laser & Optics Century Co., Ltd., Shanghai, China) coupled into multimode fiber. A longpass filter (BLP01-405, Semrock, Rochester, NY) was used to reject backreflected laser light. The emission filter was a longpass filter with a cutoff at ~560 nm (BLP01-561R, Semrock). Real-time (30 frames per second) video was recorded using custom image acquisition software. Given that the fluorescence emission covered roughly half of the visible spectrum, white light video of the surgical field was not recorded during intraoperative imaging.

#### Animals

All procedures were approved by the Institutional Animal Care and Use Committee (IACUC) at GE Global Research. Male CD-1 mice ranging in body weight from 25–30 g were purchased from Charles River Laboratories (Wilmington, MA) and housed at 22–23 °C on a 12 h light/dark cycle. Mice were maintained on Prolab RMH 3500 mouse chow (LabDiet Framingham, MA) and water *ad libitum*. On the day of the experiment, mice were anesthetized using 2–4 % isofluorane and given a single tail vein injection of either GE3111 in formulation or formulation excipients alone. The mice were then returned to the home cage until the designated time-point for imaging.

#### Formulation of GE3111 for Intravenous Administration

GE3111 was prepared for intravenous (IV) administration by dissolving in a buffer containing 0–0.5 % DMSO (Sigma D8418), 10–35 % propylene glycol (Fisher P355-1), 1–35 % polyethylene glycol (PEG-300; Sigma 202371), 0–30 % 2-hydroxypropyl-β-cyclodextrin (2-HPβCD, Sigma H5784), and 29.5–58.5 % sterile water (Sigma W3500). The IV formulation was brought to a final pH of 4.5 using 1M HCl. No preservative system was used as formulated doses were injected on the same day. Complete solubility of the agent in the formulation mixture was verified using (1) visual observation for particulates, (2) centrifugation (5 min, 10,000 × g) followed by observation, (3) dissolution in a physiologically relevant buffer (e.g., Sorenson's phosphate buffer) followed by visual observation and UV/Vis analysis, and (4) assessment of sedimentation and particle size using DLS.

#### Dosing and Kinetics

The dose–response and kinetics for GE3111 was determined in adult male CD-1 mice. In the dose–response study, each animal received a single dose of GE3111 4 h prior to imaging of key nerves. Doses of GE3111 in this study ranged from 0.46 to 16.67 mg/kg. Control mice were given a single injection of the IV formulation (vehicle only) and measured to determine background fluorescence. Post-processing of imaging data included line profile analysis to determine the fluorescence maxima of nerves and adjacent muscle and adipose tissue sample. The fluorescence maxima were measured in two regions of each nerve and surrounding tissue to display the average nerve-to-muscle ratio (N:M). Three mice at each dose were evaluated.

For the kinetics study, each mouse received a single injection of 3.77 mg/kg GE3111 and was euthanized at 1, 2, 3, 4, 12, and 24 h post-injection. Key nerves were then dissected and imaged. Control mice used were given a single injection of IV formulation only. Three mice at each time-point were evaluated.

## Results

### Synthesis and *In Vitro* Properties of GE3111

GE3111 was synthesized using a more direct and versatile methodology compared to what was previously described for BMB, GE3081, and GE3082 [20, 31]. This method consisted of a tandem Heck coupling followed by a Horner–Wittig olefination using commercially available bromoaldehyde for the middle ring and readily available building blocks for the terminal rings (Fig. 1).

GE3111 has a molecular weight of 405 g/mole and a logD value at pH 7.4 of 4.5, which is half a log unit lower than that of GE3082 (Table 1). The maximum solubility in the IV formulation buffer, consisting of 58.5 % distilled water, 30 % 2-HPβCD, 10 % propylene glycol, 1 % PEG-300, and 0.5 % DMSO, was estimated using UV/Vis spectroscopy. Under the same conditions, the maximum solubility of GE3111 was about six times more than GE3082 (Table 1).

**Table 1:**
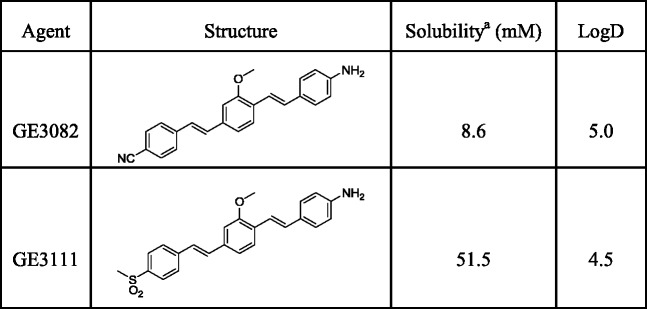
Physical characterization of GE3111 and GE3082

^a^Maximum solubility when formulated in 58.5 % distilled water, 30 % 2-HPβCD, 10 % propylene glycol, 1 % PEG-300, and 0.5 % DMSO

**Fig. 2:**
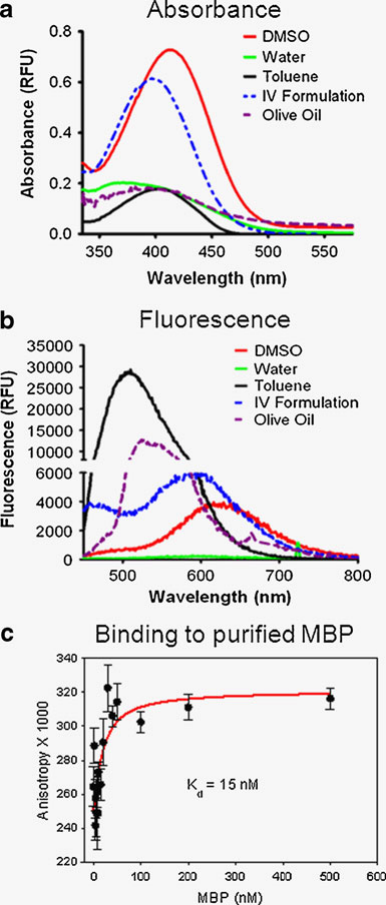
*In vitro* properties of GE3111. The absorbance (**a**) and fluorescence emission spectra (**b**) are shown in DMSO, water, toluene, IV formulation (58.5 % distilled water, 30 % 2-hydroxypropyl-β-cyclodextrin, 10 % propylene glycol, 1 % PEG-300, and 0.5 % DMSO), and olive oil. (**c**) Binding of GE3111 to purified native myelin basic protein is measured by fluorescence polarization. Unbound GE3111 has an average anisotropy of 0.250. Incubation of 100 nM GE3111 with an increasing concentration of MBP resulted in an increase in anisotropy saturating at 0.321. The calculated binding affinity (*K*_d_) is about 15 nM.

To investigate how local environment affects optical properties, we recorded the spectroscopic properties in a series of solvents varying in polarity. In general, GE3111 showed a bathochromic shift in fluorescence emission spectra with increasing solvent polarity, similar to GE3082 (Table 2, Fig. 2a, b). Moreover, a significant increase in quantum yield was observed with decreasing solvent polarity. For example, the more polar solvent DMSO exhibited a ~100 nm red-shift in fluorescence emission and a five-fold decrease in quantum yield compared to the least polar solvent, toluene.

**Table 2:** Solvent dependence of the optical properties of GE3111

Solvent (D.C.)^a^	*ε* (M^-1^ cm^-1^)	Ex max (nm)	Em max (nm)	QY (%)
Water (80)	19,680	362	590	0.1
DMSO (46.7)	72,820	412	629	1.0
Olive oil (3.1)	17,700	382	521	4.8
Toluene (2.4)	17,680	402	527	5.3
IV formulation^b^ (not determined)	60,840	396	594	1.5

*ε* molar extinction coefficient, *Ex max* excitation maximum wavelength, *Em max* emission maximum wavelength, *QY* quantum yield

^a^Dielectric constant, values from http://macro.lsu.edu/ and http://orioninstruments.com/html/tools/dielectric.aspx

^b^Contained 58.5 % distilled water, 30 % 2-HPβCD, 10 % propylene glycol, 1 % PEG-300, and 0.5 % DMSO

### Specificity of GE3111 for Purified Myelin Basic Protein

Although myelin has been suggested as the binding target for GE3082 [20], little is known as to the exact specificity of these fluorophores for MBP, a major protein component of myelin. The affinity of GE3111 to native MBP was determined using a fluorescence polarization-based binding assay that measured the anisotropy of a fixed concentration of GE3111 (100 nM) either in the absence or presence of increasing concentrations of purified native MBP. Unbound GE3111 in buffer had an anisotropy value of 0.250. Incubation of GE3111 with increasing concentrations of MBP caused a corresponding increase in the anisotropy values that saturate out at 0.321 (Fig. 2c), suggesting slower rotation of GE3111 due to a binding interaction between GE3111 and MBP. The anisotropy data were mathematically fitted, resulting in a dissociation constant (*K*_d_) of 15 ± 10 nM, indicating a strong affinity of the fluorophore for MBP. Details of the binding assay will be published elsewhere (Bajaj et al., manuscript in preparation).

### *In Vivo* Fluorescence Imaging

Initial nerve *in vivo* imaging was performed using a CRI-Nuance multispectral camera to assess the overall spectral differences among tissue types. Following *in vivo* characterization of GE3111 in mice, the overall feasibility of real-time nerve visualization in an intraoperative setting was demonstrated by recording live video during a surgical dissection of a GE3111-labeled mouse nerve using a compact instrument built in-house.

**Fig. 3:**
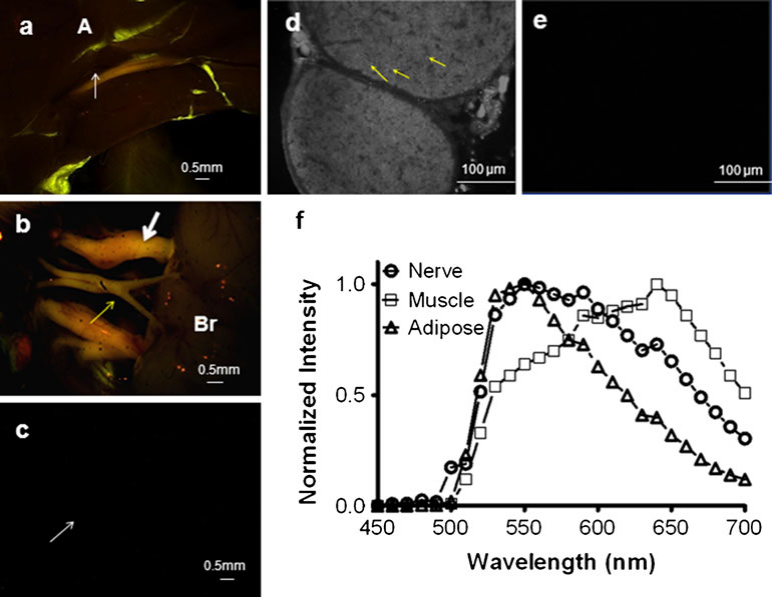
Multispectral imaging of GE3111 *in vivo*. Images were recorded 4 h post-IV administration of a 16.67 mg/kg dose. Representative fluorescence images of a mouse sciatic nerve (**a**) and optic and trigeminal nerves (**b**) are shown. Nerve location in each image is indicated by an *arrow* (*light white* for sciatic nerve, *heavy white* for trigeminal nerves, *yellow* for optic nerves), and adjacent tissue structures such as adipose (*A*) and brain (*Br*) are annotated. Mice receiving only a single injection of formulation excipients only (no GE3111) were included as control (**c**). A cross-sectional fluorescence microscopy image of the sciatic nerve depicts binding of GE3111 to the myelin sheath (examples are indicated by *yellow arrows*) surrounding the nerve axon (**d**). A cross-sectional image of a sciatic nerve of a control animal is completely dark (**e**). Normalized spectra of nerve, muscle, and adipose are shown to illustrate spectral separations between tissue types (**f**).

GE3111 fluorescence was observed in mouse nerves containing myelin such as brachial plexus, trigeminal, optic, sciatic, facial, femoral, vagus, phrenic, median, radial, supracapular, and laryngeal nerves as well as the brain and spinal column. Fluorescence was also observed in adipose tissue. Representative images collected from sciatic nerves, trigeminal, and optic nerves labeled *in vivo* with either formulation buffer-only (control) or GE3111 are shown in Fig. 3a–c. Cross-sectional analysis of the control and GE3111-labeled sciatic nerve is shown in Fig. 3d, e. Specific labeling of the myelin sheath surrounding the nerve and not the surrounding connective tissue was observed with GE3111. Multispectral imaging showed that while the fluorescence intensity in adipose tissue was high, the emission wavelength of GE3111 in adipose tissue was blue-shifted (emission maximum at 550 nm, Fig. 3f) compared to that in peripheral and central nervous tissue (emission maximum at 590–600 nm). Visually, the nerves appeared red-orange, while the adipose tissue appeared yellow-green. The surrounding muscle tissue was dark.

#### Dose and Kinetics of GE3111

**Fig. 4:**
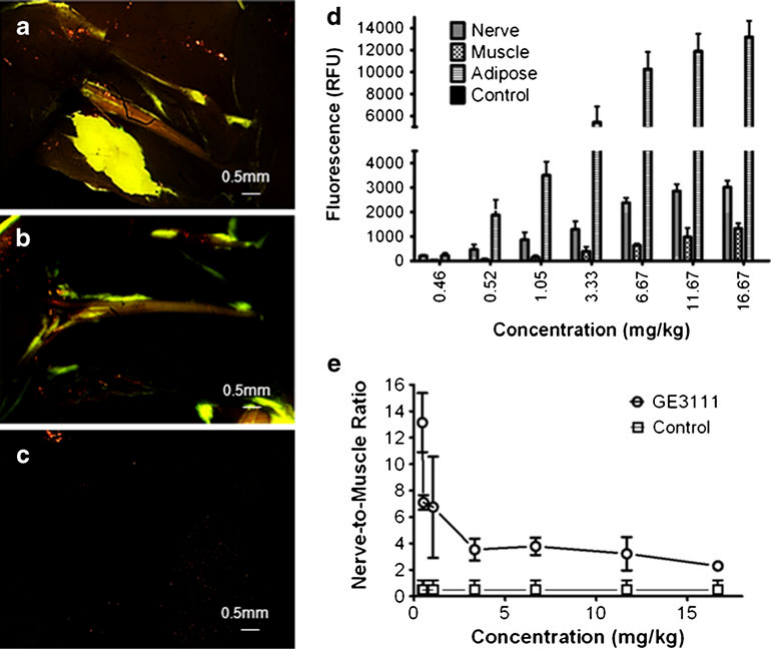
Dose and tissue specific fluorescence in mice following IV administration of GE3111 formulated with 58.5 % distilled water, 30 % 2-HPβCD, 10 % propylene glycol, 1 % PEG-300, and 0.5 % DMSO at the following concentrations (mg/kg): 16.67, 11.67, 6.67, 3.33, 1.05, 0.52, 0.46, and control/formulation excipients only. Sample images of mouse sciatic nerves collected 4 h following a dose of 11.67 mg/kg (**a**), 3.33 mg/kg (**b**), or formulation excipient only (**c**) are shown. The area under the curve for the fluorescence spectrum acquired in sciatic nerve, adjacent muscle, and adipose is calculated and reported as fluorescence (**d**); *n* = 3 mice per group. The nerve-to-muscle ratio (mean ± SD) was calculated using the total fluorescence in the sciatic nerve as compared to that of adjacent muscle tissue (**e**).

The dose–response and kinetics for GE3111 were evaluated in mice. In the dose–response study, mice received a single injection of GE3111 in concentrations ranging from 0.46 to 16.67 mg/kg formulated in 58.5 % distilled water, 30 % 2-HPβCD, 10 % propylene glycol, 1 % PEG-300, and 0.5 % DMSO. Control mice were given a single injection of IV formulation buffer only to assess background fluorescence. Four hours after injection, mice were euthanized, and the sciatic nerves were exposed by removal of biceps femoris. Example images of sciatic nerves with different doses of GE3111 are shown in Fig. 4a–c. Post-processing of images using line profile analysis was performed to determine fluorescence maxima in the sciatic nerve and adjacent muscle and adipose. Fluorescence emission intensity increased in the sciatic nerve up to a concentration of 6.67 mg/kg. There was no significant change in fluorescence intensity in concentrations greater than 6.67 mg/kg, suggesting a saturation of binding to MBP at these concentrations. However, fluorescence emission in adipose tissue showed no indications of saturation consistent with lack of specific binding target (Fig. 4d). Minimal fluorescence was seen in adjacent muscle tissue. Finally, all concentrations measured in the dose–response relationship exhibited a nerve-to-muscle ratio (N:M) greater than the control (Fig. 4e).

**Fig. 5:**
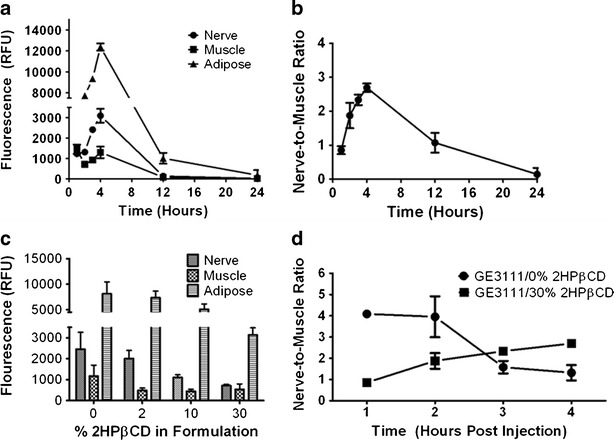
The kinetics and overall effect of formulation on tissue-specific fluorescence. A single injection of 3.77 mg/kg GE3111 formulated in 58.5 % distilled water, 30 % 2-HPβCD, 10 % propylene glycol, 1 % PEG-300, and 0.5 % DMSO was given 1, 2, 3, 4, 12, and 24 h prior to imaging. (**a**) The area under the curve for the fluorescence spectrum acquired in sciatic nerve, adjacent muscle, and adipose is calculated and reported as fluorescence (*n* = 3 mice per group). (**b**) The nerve-to-muscle ratio was then calculated using the total fluorescence reported in (**a**) as compared to adjacent muscle tissue. In a separate experiment, mice were given a dose of 3.77 mg/kg GE3111 in formulations varying in 2-HPβCD concentration (e.g., 0 %, 2 %, 10 %, or 30 %), and the images were collected 1 h post-IV administration of GE3111 (**c**). Tissue specific fluorescence in the sciatic nerve, adjacent muscle, and adipose (*n* = 3 mice per group) is shown. In (**d**), nerve-to-muscle ratios of 3.77 mg/kg GE3111 formulated in either 0 % or 30 % 2-HPβCD and given at 1, 2, 3, and 4 h post-injection are shown (*n* = 3 mice per group).

Based on the dose–response study, a theoretical half maximal dose of GE3111 was calculated to be 3.77 mg/kg. This dose was then used to assess the kinetics of GE3111 *in vivo* by measuring the fluorescence in nerve at the following time-points: 1, 2, 3, 4, 12, and 24 h post-IV injection. Control mice received a single injection of formulation buffer only. Maximum muscle fluorescence was observed at 1 h post-IV injection, resulting in a nerve to muscle ratio of 0.95 ± 0.12 (Fig. 5a, b). The overall fluorescence in muscle decreased consecutively following the 1-h time-point. As muscle fluorescence decreased, the overall fluorescence in nerve and adipose increased, reaching a maximum at 4 h post-IV injection (N:M = 3.1 ± 0.10; Fig. 5a, b). No fluorescence was seen in control mice (data not shown). After 4 h, fluorescence emission decreased dramatically in all tissues. At 12 h post-IV injection, no visible fluorescence was present in nerve and adjacent muscle. However, minimal fluorescence was still visible in adipose tissue. This could be due to the presence of higher fluorescence intensity in adipose tissue to begin with or slower clearance from adipose tissue due to its poorly perfused nature as compared to other peripheral tissues [32]. No animals were used for more than one time-point to prevent aberrant data resulting from anesthesia and/or surgically induced changes in pharmacokinetics and pharmacodynamics.

GE3111 reached a maximum fluorescence in nervous tissue at 4 h post-IV injection as shown previously (Fig. 5a, b). However, modifications in formulating the buffer components can be utilized to either increase or decrease the overall kinetics *in vivo*. In these experiments, 3.77 mg/kg of GE3111 was administered in varied formulation protocols. These formulation protocols included usage of concentrations of 2-HPβCD ranging from 0 % to 30 % and increasing concentrations of propylene glycol and PEG-300, used to compensate for decreases in solubility. No animal was used for measurements deriving from more than a single formulation at any set time-point. At 1 h post-IV injection, fluorescence emission was highest in the 0 % and 2 % 2-HPβCD formulation compared with values obtained using 10 % and 30 % 2-HPβCD (Fig. 5c). GE3111 formulated in 30 % 2-HPβCD had maximal N:M at 4 h post-IV injection, but had a maximal N:M at 1 h post-IV injection when formulated at 0 % 2-HPβCD (Fig. 5d). These results suggest that following IV injection, the inclusion complex formed between GE3111 and 2-HPβCD could limit the initial efficacy of the drug. Furthermore, the effects of inclusion complex appear to saturate at approximately 10 % 2-HPβCD as indicated by the lack of further effects in concentrations above 10 %.

#### Image-Guided Surgery Using the Compact Instrument

**Fig. 6:**
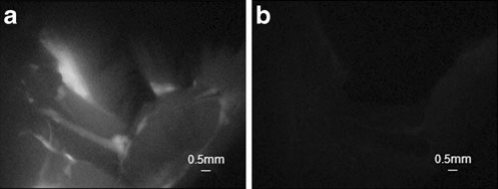
Individual frames extracted from real-time video recorded during an open surgery using a compact instrumentation built in-house. A dose of 3.33 mg/kg GE3111 was administered to adult male CD-1 mice. The compact instrumentation was positioned immediately above the mouse. Sciatic nerve fluorescence images were acquired 4 h post-IV administration of the fluorophore (**a**) and compared with images from a naïve mouse receiving a control/formulation excipient only (**b**).

Following *in vivo* characterization of GE3111 in mice using the commercial, small animal imaging instrumentation, we tested the feasibility of our modified compact instrument [30] for use during open surgical procedures. A dose of 3.33 mg/kg was administered to adult male CD-1 mice. The imaging system was positioned immediately above the mouse, allowing for live image capture, while an adjacent monitor displayed real-time video during the procedure. Using less than 2.5 mW/cm^2^ excitation power of laser illumination at 405 nm laser, high brightness images of the emitted fluorescence were captured. Without administration of GE3111, no fluorescence was visible in nervous tissue. Figure 6 shows still images of control and GE3111-labeled tissue.

## Discussion

Nerve trauma is a major cause of morbidity associated with several surgical procedures and can lead to post-surgical complications that could have deleterious effect on the patient's quality of life [1, 3, 7, 33–35]. A method to better visualize nerves prior to injury could improve patient outcome by reducing the risk of nerve damage.

Fluorescently conjugated nerve contrast agents have been described, including a protein fragment for labeling retrograde transport in the nerves following intramuscular administration [36], as well as peptides that target the connective tissue in the epineurium and endoneurium [39]. GE3111 and the analogs that we reported on previously [20] are small molecule distyrylbenzene dyes, which are capable of crossing the blood–brain barrier (BBB) and blood–nerve barrier (BNB) effectively. In general, small molecules are less costly to produce and may be engineered by chemical modifications to confer more appropriate characteristics such as improved pharmacokinetics, lipophilicity, and target affinity. Thus, they constitute a working base for the development of promising candidates for use in image-guided surgery.

A number of key traits must be met for the optimization of contrast agents for *in vivo* imaging of nerves through myelin. The fluorophore has to selectively target a component of myelin and must be capable of penetrating the BNB, which is similar in both feature and function to the BBB. Most molecules that do cross the BBB have high lipid solubility as measured by their logD's, with values between 1 and 4 as ideal [38]. Because of this, some degree of non-specific partitioning to adipose tissue could be expected. Additionally, the molecular weight should be less than 600–700 g/mole, with 400 g/mole as ideal [39]. Therefore, the myelin-targeting moiety has to be inherently fluorescent because conjugating it to a dye could significantly increase its molecular weight beyond the desirable range.

GE3111 has a molecular weight of 405 g/mole and binds to a major component of myelin, MBP. Its optical properties *in vitro* support previous findings which suggested that optical properties of molecules which contain both electron donating and withdrawing groups in the same molecule can be sensitive to the local environment [20]. Its logD of 4.5 is not yet ideal, but is better than that of GE3082. The improvement in aqueous solubility resulted in easier formulation using more clinically relevant excipients and less disperse solution once formulated.

GE3111 crossed the BNB and BBB after a single IV injection and localized to central and peripheral nerves. Its kinetics of maximal uptake in the nerve was adjustable, depending on the amount of 2-HPβCD in the formulation buffer. The maximal N:M was achieved 4 h post-injection and 1 h post-injection when formulated in 30 % and 0 % 2-HPβCD, respectively. It is possible that the formation of inclusion complexes between GE3111 and the internal pore of 2-HPβCD, a cyclic oligosaccharide, can slow the kinetics of the drug distribution *in vivo* by reducing the free-to-bound drug ratio post-IV injection [40].

Under multispectral imaging, the nerve fluorescence appeared in a different color than the adipose tissue fluorescence, which also exhibited higher fluorescence intensity. One explanation for the increase in adipose tissue fluorescence intensity is the dependence of agent optical properties with local environment. Adipose tissue is highly non-polar, with the majority of fatty acid content comprising of oleic, palmitic, and linoleic acid [41], similar to olive oil. The effect of solvent polarity (Table 2) on quantum yield and fluorescence emission could help explain the observed effect of GE3111 in adipose tissue.

The dosing and kinetics studies were performed using a commercial, small animal imaging instrument. Once the *in vivo* spectroscopic properties were determined, a compact, intraoperative imaging instrument was adapted accordingly for real-time imaging of GE3111-labeled nerves. The modifications on the compact device were focused on optimization for usage with GE3111. Through the use of consumer grade cameras and fiber optic delivery of laser light, we have reduced the size (~2 kg) and cost (~$10,000) of imaging instrumentation, as compared to previous clinical optical instrumentation on the order of $100,000.

Several recent works have presented the merits of fluorescence imaging in the near infrared (NIR), namely, clear spectral separation of the fluorescence and the color channels, reduction of autofluorescence, and increased penetration depth relative to excitation in the ultraviolet/visible [42, 43]. These benefits, coupled with the development of NIR fluorophores, have resulted in successful deployment of NIR instrumentation for preclinical and clinical surgical imaging. In this paper, we have described another viable approach for fluorescence imaging using visible dyes. Although illuminating the tissue at 405 nm excites autofluorescence, the relatively large Stokes shift of GE3111 allows for efficient spectral separation of the dye fluorescence from background. Additionally, the peak emission wavelength is in the green, corresponding to the peak responsivity of conventional silicon detectors, and thus higher sensitivity detection, as much as 2–3 times higher than NIR wavelengths. A potential disadvantage of visible fluorescence is that it impedes the concomitant detection of color video using white light. One way to address this is to alternate between the white light and fluorescence channels, creating a dual-mode instrument through time multiplexing. The excitation wavelength at 405 nm can then readily be removed from the optical path with minimal impact on the color video channel [44].

### Conclusions

Selective contrast agents for nerve imaging, coupled with a practical implementation of instrumentation, represent a step towards clinical translation of fluorescence image-guided surgery for prevention of nerve damage. Future initiatives will focus on expanding the understanding of pharmacologic activity of GE3111, such as determining its concentration in key tissue targets through quantitative *in vivo* biodistribution, identifying potential metabolites, and assessing its toxicology profile using cell culture. We will also advance the imaging instrumentation towards dual-mode imaging and minimally invasive surgical procedures.

## Electronic supplementary material

Below is the link to the electronic supplementary material.

ESM 1 (PDF 70 kb)
