# Supplementary material for: Intraoperative Fluorescence Imaging of Peripheral and Central Nerves Through a Myelin-Selective Contrast Agent
Source: Mol Imaging Biol. 2012 Apr 10;14(6):708–17. doi: 10.1007/s11307-012-0555-1 (PMC3492698; doi:10.1007/s11307-012-0555-1)
Supplement: Supplementary file 1 — (PDF 70 kb) [file 11307_2012_555_MOESM1_ESM.pdf]

Supplementary Material

Molecular Imaging and Biology

Intraoperative Fluorescence Imaging of Peripheral and Central Nerves through Myelin-Selective Contrast Agent

Victoria E. Cotero, Tiberiu Siclovan, Rong Zhang, Randall L. Carter, Anshika Bajaj, Nicole E. LaPlante, Evgenia Kim, Daniel Gray, V. Paul Staudinger, Siavash Yazdanfar, and Cristina A. Tan Hehir*

GE Global Research, One Research Circle, Niskayuna, NY 12309

*Address correspondence to tanhehir@research.ge.com

A. Synthesis of GE3111 (1-methylsulfonyl-4-[(1E)-2-[4-[(1E)-2-[4-aminophenyl] ethenyl]-3-methoxyphenyl] ethenyl]-benzene)

***(E)-tert-butyl 4-(4-formyl-2methoxyphenylethenyl)phenyl carbamate* (2):** A solution of 4-Bromo-3-methoxybenzaldehyde (106 mg, 0.49 mmol), tert-butyl 4-vinylphenylcarbamate (141 mg, 0.64 mmol), palladium acetate (17 mg, 0.074 mmol), TPPTS (70 mg, 0.12 mmol), and potassium carbonate (205 mg, 1.48 mmol) in water/DMF (1:1; 2.5mL) was stirred for 3 h at 95ºC. The solution was then diluted with ethyl acetate and extracted three times with additional ethyl acetate. The combined organic layers were poured over saturated brine and immediately dried over Na_2SO_4. The crude product was purified by medium pressure liquid chromatography (MPLC) using hexane-ethyl acetate 0 to 25% gradient to give the desired intermediate as a yellow solid (122 mg, 70% yield). Mass spectroscopy (MS) electrospray ionization (ESI^+): 395 ($M+CH_3CN+H^+$); 354 ($M+H^+$). ^1H NMR (400 MHz, $CDCl_3$) δ 9.95 (s, 1H), 7.73 (d, J = 7.83 Hz,

1H) 7.50 (d, J = 8.84 Hz, 1H) 7.45 (m, J = 15.66 Hz, 2H), 7.38 (m, J = 14.4 Hz, 3H), 7.23 (d, J = 15.53 Hz, 1H), 6.53 (d, J = 15.7 Hz, 1H), 3.96 (s, 3H), 1.53 (s, 3H).

Diethyl (4-methylsulfonyl)benzyl phosphonate **(3)*:*** 4-Methylsulfonylbenzyl bromide (5 g, 20 mmol) was dissolved in triethyl phosphite (11.2 ml, 80 mmol) and the solution was gradually heated to 100°C. Gas evolution began at 80°C and continued at a moderate rate afterwards. The mixture was stirred for 2 h at 100°C, at which point GC-MS analysis indicated complete and clean conversion to the desired product. The excess triethyl phosphite was removed under reduced pressure and the remaining traces of this reagent were stripped with a stream of dry nitrogen. The product thus obtained (colorless oil, 6g, 98%) was used in the next step without further purification. MS (EI^+): 306 (23%; M^+), 278(25%, M-C_2H_4), 263, 250(18%), 227, 199, 183, 170(90%), 153, 139, 124(82%), 107(100%), 90.

1-methylsulfonyl-4-[(1E)-2-[4-[(1E)-2-[4-tert-butoxycarbonylaminophenyl] ethenyl]-3-methoxyphenyl] ethenyl]-benzene **(4)*:*** A solution of potassium-tert-butoxide (t-BuOk; 1.94 g, 17.29 mmol) in THF was added slowly to a solution of diethyl (4-methylsulfonyl) benzyl phosphonate (4.49 g, 14.67 mmol) in tetrahydrofuran (THF; 50 mL) via cannula. The solution was stirred for 5 min and then promptly transferred, under nitrogen (N_2), to a solution of aldehyde 2 (Fig. 1; 5.08 g, 14.39 mmol) in THF (25 mL). The resulting solution was heated to 64°C and stirred for 2 h while under constant N_2. Liquid chromatography-mass spectroscopy (LC-MS) analysis indicated complete conversion of the aldehyde.

The solution was allowed to cool prior to addition of gaseous CO_2 (from dry ice) to use above the liquid layer for 30 min allowing for precipitation of salts in the flask. The supernatant was decanted from the mass of precipitated salts, which were triturated three times with 100 mL dichloromethane. The organic phases were combined and concentrated under pressure to a final

volume approximately ~1/5th of its original volume. The product was then filtered and dried at room temperature under a vacuum to yield an intermediate of fine yellow crystals (5.96 g, 82% yield). The intermediate was sufficiently pure to be used in the next step without further purification. Mass spectroscopy (MS) electrospray ionization (ESI^+):528($M+Na^+$); 505 (M^+).

1-methylsulfonyl-4-[(1E)-2-[4-[(1E)-2-[4-aminophenyl] ethenyl]-3-methoxyphenyl] ethenyl]-benzene **(5) GE3111:** 33 mL of TFA was added to a solution of *1-methylsulfonyl-4-[(1E)-2-[4-[(1E)-2-[4-tert-butoxycarbonylaminophenyl] ethenyl]-3-methoxyphenyl] ethenyl]-benzene* (compound 4; 3.5 g, 6.93 mmol) in 133 mL dichloromethane (containing 40 ppm amylene), at 0°C. The solution was then allowed to gradually warm up to room temperature. LC-MS analysis at 30 min indicated a complete conversion to the product. The solvent was removed under reduced pressure at 20°C. Finally, the residual TFA was removed under a high vacuum and the resulting reddish solid was purified by reverse-phase chromatography (Waters XBridge 250x50 mm, 10 µm) using water-CH_3CN gradient (50% to 60% CH_3CN) with 0.1% TFA, 1 g/injection. The final product yield of GE3111 was 3.52 g as a salt with 0.7 equivalents of TFA. The fluorophore was initially synthesized as a TFA salt to improve stability for prolonged storage. However, free base dye was prepared via aqueous workup ($NaHCO_3$/ dichloromethane), yielding the required dye in better than 99.9% purity. Mass spectroscopy (MS) electrospray ionization (ESI^+):528($M+Na^+$); 505 (M^+).447($M+CH_3CN+H^+$); 406($M+H^+$).

The purity of the final compound was determined using NMR spectroscopy. ^{1}H-NMR (CD_2Cl_2): δ 7.95 (d, 2H, J=8.4Hz) 7.75Hz (d, 2H, J=8.4Hz) 7.56 (d,1H, J=8.4Hz) 7.32(m, 3H) 7.24(d, 1H, J=16.4Hz) 7.18(m, 2H) 6.62(d, 2H, J=8Hz) 3.92(s,3H) 3.23 (s,3H). ^{13}C-NMR (CD_2Cl_2): δ 156.59, 146.47, 145.79, 135.3, 134.09, 130.93, 129.65, 128.15, 127.95, 127.76, 127.64, 127.56, 127.48, 127.04, 127.01, 125.72, 124.93, 120.7, 119.94, 118.42, 114.63, 108.86,

55.24, 44.38. The presence of fluorescent impurities was determined using HPLC with attached fluorescence detection at the following setting: excitation: 375 nm, emission: 470 nm. Removal of traces of fluorescent impurities was achieved through a final purification by reverse phase chromatography, eluting with water-acetonitrile gradient containing 0.1% v/v TFA.

B. Dynamic light scattering (DLS): DLS spectroscopy was used to measure polydispersity of GE3111 and GE3082 in a selected formulation buffer (58.5% distilled water, 30% 2-hydroxypropyl-β-cyclodextrin, 10% propylene glycol, 1% PEG-300, and 0.5% DMSO). Each fluorophore was prepared at a concentration of 4.8 mM in solution and duplicate measurements were taken for Z-average diameter and polydispersity indices (PdI).

Measurements were collected on a Malvern HPPS500 spectrometer (Worcestershire, UK). Samples were measured in a low volume disposable cuvette at 25 ºC, at an angle of 173. GE3111 had a significantly smaller Z-average, as compared to GE3082 (Table 1S). This was supported by the PdI values suggesting that GE3082 in solution was considerably more disperse than GE3111.

Table 1S: DLS Spectroscopy measurements

Agent	Z-average (nm)	Polydispersity Index
GE3082	7830 +/-817	0.774 +/-0.141
GE3111	2210 +/-75	0.298 +/-0.088
